# 9-aminoacridine Inhibition of HIV-1 Tat Dependent Transcription

**DOI:** 10.1186/1743-422X-6-114

**Published:** 2009-07-24

**Authors:** Irene Guendel, Lawrence Carpio, Rebecca Easley, Rachel Van Duyne, William Coley, Emmanuel Agbottah, Cynthia Dowd, Fatah Kashanchi, Kylene Kehn-Hall

**Affiliations:** 1 The George Washington University, Department of Microbiology, Immunology, and Tropical Medicine, 2300 I Street, NW, Washington, DC 20037, USA; 2 The George Washington University, Department of Chemistry, Washington, DC 20037, USA

## Abstract

As part of a continued search for more efficient anti-HIV-1 drugs, we are focusing on the possibility that small molecules could efficiently inhibit HIV-1 replication through the restoration of p53 and p21WAF1 functions, which are inactivated by HIV-1 infection. Here we describe the molecular mechanism of 9-aminoacridine (9AA) mediated HIV-1 inhibition. 9AA treatment resulted in inhibition of HIV LTR transcription in a specific manner that was highly dependent on the presence and location of the amino moiety. Importantly, virus replication was found to be inhibited in HIV-1 infected cell lines by 9AA in a dose-dependent manner without inhibiting cellular proliferation or inducing cell death. 9AA inhibited viral replication in both p53 wildtype and p53 mutant cells, indicating that there is another p53 independent factor that was critical for HIV inhibition. p21WAF1 is an ideal candidate as p21WAF1 levels were increased in both p53 wildtype and p53 mutant cells, and p21WAF1 was found to be phosphorylated at S146, an event previously shown to increase its stability. Furthermore, we observed p21WAF1 in complex with cyclin T1 and cdk9 *in vitro*, suggesting a direct role of p21WAF1 in HIV transcription inhibition. Finally, 9AA treatment resulted in loss of cdk9 from the viral promoter, providing one possible mechanism of transcriptional inhibition. Thus, 9AA treatment was highly efficient at reactivating the p53 – p21WAF1 pathway and consequently inhibiting HIV replication and transcription.

## Introduction

HIV-1 infection results in the alteration of numerous host factors and signaling cascades [1]. In particular, it has been demonstrated that the p53 pathway plays an important role in HIV-1 infection [2,3]. p53 is critical for protecting the integrity of the genome through regulating apoptosis [4-9] and the cell cycle, at both G1/S [10-14] and G2/M checkpoints [15-19]. Wild-type p53 has the ability to be a potent suppressor of HIV-1 Tat transcriptional activity [20,21], whereas mutant p53 can activate HIV-1 transcription [22,23]. An RGD-containing domain of Tat protein, Tat (65-80), was shown to play an important role in regulating the proliferative functions of a variety of cell lines, including a human adenocarcinoma cell line, A549. p53 activity was greatly reduced when cells were treated with Tat-(65–80) [24]. On the other hand, Tat efficiently inhibits p53 transcriptional activity through blocking K320 acetylation [25]. These above observations are at least partially explained by the discovery that Tat binds directly to p53 through the p53 dimerization domain [26]. A model has been suggested where p53 could become inactivated in HIV-1 infected cells through binding to Tat and subsequently losing its ability to transactivate its downstream target gene p21WAF1 [27]. While the interplay between p53 and HIV-1 Tat has been clearly demonstrated *in vitro *by a number of researchers, the *in vivo *interaction is less clearly defined and requires further analysis. Collectively, these observations indicate the possible role of p53 in the control of HIV-1 replication patterns and proviral latency [22].

One of the most well characterized transcriptional targets of p53 is the p21WAF1 gene. p21WAF1 was simultaneously characterized by a number of different researchers; it has been described as a target of p53 transactivation, a cyclin/cyclin-dependent kinase (cdk) inhibitor and a protein that is expressed in senescent fibroblasts [28-31]. In addition to its most well-known role as a cdk inhibitor (CKI) that can lead to cell cycle arrest, p21WAF1 is also well recognized to be involved in a variety of other physiological functions. These include the promotion of differentiation as well as the imposition of cellular senescence [32,33]. The anti-proliferative functions of p21WAF1 are associated with its ability to bind to PCNA and block DNA synthesis. Nuclear p21WAF1 also participates in regulating several transcriptional responses, as well as regulating DNA methylation [34,35]. While in the cytoplasm p21WAF1 also has important pro-proliferative and survival functions including promoting the formation of cyclin D/cdk4, 6 complexes [36-38] and negatively regulating Fas-mediated apoptosis through the inactivation of procaspase 3 [34,35].

As the regulation of the p53 and p21WAF1 pathways by HIV-1 infection has become a point of great interest, it might be possible to combat HIV-1 infection through the restoration of the p53 and p21WAF1 pathways using small molecules, such as 9-aminoacridine (9AA). 9AA was originally identified as an anti-bacterial agent, but more recently has gained notice as a potential treatment for cancer, viral, and prion diseases [39-41]. Enthusiasm for 9AA was initially dampened due to observed toxicity that was suggested to be due to DNA intercalating properties and possible topoisomerase II poisoning [42-44]. However, later studies have demonstrated that 9AA can be utilized in a selective manner, especially for virally infected cells. In a 2008 study, up to 20 μM 9AA was utilized with no toxicity observed in uninfected cell lines or PBMCs [45]. In addition, an independent group demonstrated that 9AA treatment did not induced phosphorylation of histone H2A.X or activate the DNA response kinases ATM or ATR, all of which are indicators of DNA damage [41]. 9AA was not found to cause DNA damage by poisoning topoisomerase II as had been previously suggested [41]. Therefore, it appears that 9AA activates p53 through a mechanism different than DNA damage induced p53. 9AA treatment of renal carcinoma cells and HTLV-1 infected T-cells demonstrated NF-κB inhibition and p53 activation, with NF-κB inhibition being upstream of p53 activation [41,45,46]. 9AA triggered cell death is dependent on p53, as p53 siRNA blocks 9AA induced cell death [45]. More recently, Guo *et al*. demonstrated through proteomics analysis downregulation of p110γ, the catalytic subunit of the phosphoinositide 3-kinase (PI3K) family upon 9AA treatment of renal carcinoma cells [47]. Follow-up studies indicated that AKT and the mammalian target of rapamycin (mTOR) signaling were inhibited, which contributed to p110γ downregulation, and possibly p53 and NF-κB alterations.

Previously we have shown that 9AA efficiently reactivates the p53 and p21WAF1 pathways in HIV-1 infected cells [46]. Specifically, we observed increased S15 phosphorylation of p53 and increased p21WAF1 protein levels. p53-pS15 was not detected in complex with Tat, freeing p53 from Tat inhibition. Importantly, virus replication was found to be inhibited in HIV infected PBMCs by 9AA in a dose-dependent manner. Here we investigate further the mechanism of 9AA HIV-1 inhibition. We show that 9AA treatment resulted in inhibition of Tat dependent HIV-1 transcription, without inhibition of cellular proliferation. Using various 9AA derivatives we determined that the amino moiety of 9AA is critical for the observed transcriptional inhibition. We observed for the first time p21WAF1 in complex with p-TEFb (cyclin T1 and cdk9) *in vitro*, suggesting a role of p21WAF1 in HIV-1 transcription and 9AA mediated inhibition of viral transcription. Finally, we observed loss of the critical transcriptional cofactor, cdk9, from the LTR following 9AA treatment, indicating that this is one possible mechanism of 9AA mediated viral inhibition. Thus, 9AA treatment is highly efficient at reactivating p53 and p21WAF1 pathways and inhibiting HIV replication.

## Materials and methods

### Cell Culture

ACH2 and J1.1 are latently HIV-1 infected T-cell lines. ACH2, J1.1, CEM, and Jurkat cells were grown in RPMI-1640 media containing 10% fetal bovine serum (FBS), 1% L-glutamine, and 1% streptomycin/penicillin. TZM-bl cells were grown in Dulbecco's modified Eagle's medium supplemented with 10% FBS, 1% L-glutamine, and 1% streptomycin/penicillin. All cells were incubated at 37°C and 5% CO_2_.

### Small Molecule Compounds and Antibodies

9-aminoacridine was obtained from Sigma, 2-aminoacridine and 4-aminoacridine from KaironKem, acridine hydrochloride from TCI, and 4-aminoquinoline from Tyger Scientific. p21WAF1, phospho-p21WAF1 (S146), cdk2, cyclin T, and actin antibodies were obtained from Santa Cruz Biotechnology. Cdk9 antibody was obtained from Biodesign International. p53, phospho-p53 (S15), AKT (pan), phospho-AKT (S473), phospho-AKT (T308), GSK3-β (pan), phospho-GSK3-β (S9) antibodies were obtained from Cell Signaling.

### CAT Assay

Plasmids (LTR-CAT and/or CMV-Tat) were transfected by electroporation using a Bio-Rad Gene Pulser (Bio-Rad, Richmond, CA) at 960 μF and 230 volts. Two hours after transfection drug treatment was initiated. After 48 hours, cells were lysed and chloramphenicol acetyltransferase (CAT) activity was determined. Briefly, a standard reaction was performed by adding the cofactor acetyl coenzyme A to a microcentrifuge tube containing cell extract (50 ug) and radiolabeled (^14^C) chloramphenicol in a final volume of 30 μl and incubating the mixture at 37°C for 1 hour. The reaction mixture was then extracted with ethyl acetate and separated by thin-later chromatography on silica gel plates (Baker-flex silica gel thin-later chromatography plates) in a chloroform-methanol (19:1) solvent. The resolved reaction products were then detected by exposing the plate to a PhosphoImager cassette.

### Luciferase Assay

TZM-bl cells were transfected with pc-Tat (0.5 ug) using the Attractene reagent (Qiagen) according to the manufacturers' instructions. TZM-bl cells contain an integrated copy of the firefly luciferase gene under the control of the HIV-1 promoter (obtained through the NIH AIDS Research and Reference Reagent Program). The next day, cells were treated with DMSO or the indicated compound. Forty-eight hours post drug treatment, luciferase activity of the firefly luciferase was measured with the BrightGlo Luciferase Assay (Promega). Luminescence was read from a 96 well plate on an EG&G Berthold luminometer.

### Chromatin Immunoprecipitation Assay (ChIP)

ACH2 cells were treated with 2.5 uM 9AA and processed 48 hours later for ChIP. For ChIP, approximately 5 × 10^6 ^cells were used per IP. Cells were cross-linked with 1.0% formaldehyde at 37°C for 10 minutes, pelleted, washed, and cells lysed using SDS lysis buffer (1% SDS, 10 mM EDTA, 50 mM Tris-HCl, pH 8.0, one tablet complete protease inhibitor cocktail per 50 ml) on ice for 10 mins. Cells were sonicated on ice for 6 cycles to obtain an average DNA length of 500 to 1200 bp. Lysate was clarified by centrifugation at 14,000 rpm for 10 minutes at 4°C. Supernatant was then diluted 10 fold in ChIP dilution buffer (0.01% SDS, 1.1% Triton X-100, 1.2 mM EDTA, 16.7 mM Tris-HCl, pH 8.0, 167 mM NaCl) and pre-cleared with a mixture of protein A/G agarose (blocked previously with 1 mg/ml salmon sperm DNA and 1 mg/ml BSA) at 4°C for 1 hour. Pre-cleared chromatin was incubated with 10 μg of antibody at 4°C overnight. Next day, 60 μl of a 30% slurry of blocked protein A/G agarose was added and complexes incubated for 2 hours. Immune complexes were recovered by centrifugation and washed once with low salt buffer (0.1% SDS, 1% Triton X-100, 2 mM EDTA, 20 mM Tris-HCl, pH 8.0, 150 mM NaCl), twice with high salt buffer (0.1% SDS, 1% Triton X-100 2 mM EDTA, 20 mM Tris-HCl, pH 8.0, 500 mM NaCl), once with LiCl buffer (0.25 M LiCl, 1% NP-40, 1% deoxycholate, 1 mM EDTA, 10 mM Tris-HCl, pH 8.0), and once with TE buffer. Immune complexes were eluted twice with elution buffer (1% SDS, 0.1 M NaHCO3) and incubating at room temperature for 15 minutes on a rotating wheel. Cross-links were reversed by adding 20 μl of 5 M NaCl and incubating elutes at 65°C overnight. The next day, proteinase K (100 μg/ml final concentration) was added and samples incubated at 55°C for 1 hour. Samples were extracted with phenol:chloroform twice and ethanol precipitated overnight. Pellets were then washed with 70% ethanol, dried, resuspended in 50 μl of TE, and assayed by PCR. Thirty-five cycles of PCR were performed in 50 μl with 10 μl of immunoprecipitated material, 0.1 μM of primers, 0.2 mM dNTPs, and 1.0 unit of Taq DNA polymerase. Finally, PCR products were electrophoresed on 2% agarose gels and visualized by ethidium bromide staining.

### Western Blot Analysis

Cell extracts were resolved by SDS PAGE on a 4-20% tris-glycine gel (Invitrogen). Proteins were transferred to Immobilon membranes (Millipore) at 200 mA for 2 hours. Membranes were blocked with Dulbecco's phosphate-buffered saline (PBS) 0.1% Tween-20 + 3% BSA. Primary antibody against specified antibodies was incubated with the membrane in PBS + 0.1% Tween-20 overnight at 4°C. Membranes were washed two times with PBS + 0.1% Tween-20 and incubated with HRP-conjugated secondary antibody for one hour. Presence of secondary antibody was detected by SuperSignal West Dura Extended Duration Substrate (Pierce). Luminescence was visualized on a Kodak 1D image station.

### RT Assays

Supernatants from ACH2 and J1.1 cells were collected to test for the presence of virus on day 7 post 9AA treatment. Viral supernatants (10 μl) were incubated in a 96-well plate with reverse transcriptase (RT) reaction mixture containing 1× RT buffer (50 mM Tris-HCl, 1 mM DTT, 5 mM MgCl_2_, 20 mM KCl), 0.1% Triton, poly(A) (1 U/ml), pd(T) (1 U/ml), and [^3^H]TTP. The mixture was incubated overnight at 37°C, and 10 ml of the reaction mix was spotted on a DEAE Filtermat paper, washed four times with 5% Na_2_HPO_4_, three times with water, and then dried completely. RT activity was measured in a Betaplate counter (Wallac, Gaithersburg, MD).

### GST Pulldown Assays

GST tagged proteins were purified as described previously [46]. GST-p21 (1 μg), GST-p21 (N) (1 μg), GST-p21 (C) (1 μg), or GST (1 μg) proteins were added to 2 mg of CEM extracts from various cell lines and rotated overnight at 4°C. The next day complexes were washed twice with TNE_150 _+ 0.1% NP-40 and once with TNE_50 _+ 0.1%NP-40. Complexes were run on 4–20% Tris-glycine gel. Western blots were performed with anti-cdk9 (Biodesign), anti-cyclin T, and anti-cdk2 (Santa Cruz) antibodies.

### MTT Assays

Five thousand cells were plated per well in a 96-well plate and the next day cells were treated with various concentrations of compounds (1, 10, 50 μM) or DMSO. Forty-eight hours later, 10 μl MTT reagent (50 mg/ml) was added to each well and plates incubated at 37°C for 2 hours. Next, 100 μl of DMSO was added to each well and plate was shaken for 15 minutes at room temperature. The assay was read at 570 nM.

## Results

### 9AA inhibits HIV-1 Tat dependent transcription

**Figure 1:**
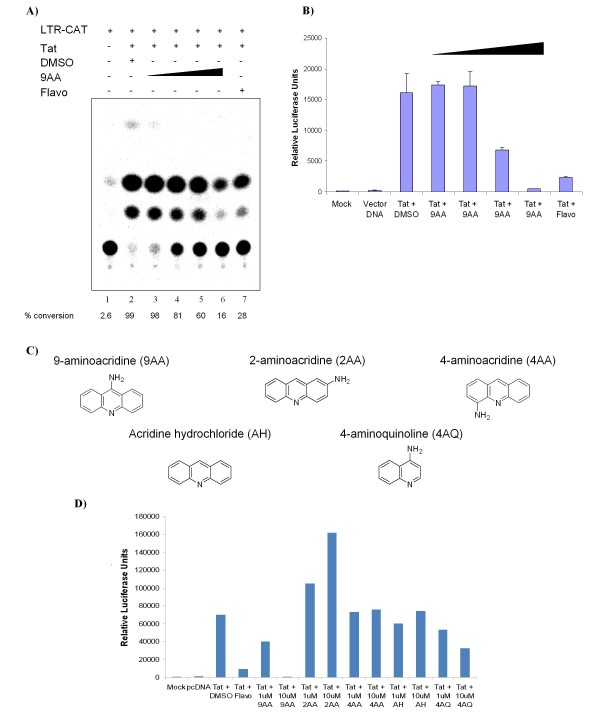
**9AA inhibits HIV-LTR transcription**. A) CEM cells were transfected with 2.5 μg HIV-LTR CAT and 0.5 μg of pc-Tat by electroporation. Twenty-four hours post-transfection, cells were treated with DMSO, 9AA (0.1, 0.5, 1, or 2.5 μM), or 100 nM Flavo (flavopiridol). Cells were harvested 48 hours post transfection and processed for CAT assays. CAT assays were performed with 4 mM acetyl CoA, 5 μl of 14C-chloramphenicol (40 mCi/mmole), 10 μl of protein extracts, and 18 μl of water. Reactions were carried out at 37°C for 30 minutes. Samples were extracted with ethyl acetate, dried, and separated by TLC. B) TZM-bl cells were transfected with 1.0 μg of Tat and treated the next day with DMSO, 9AA (0.1, 0.5, 1, or 2.5 μM), or 100 nM flavopiridol. Cells were processed 48 hours post drug treatment for luciferase assays. Assays were performed in triplicate and an average value is shown plus standard deviation. C) Structures of 9AA and derivatives. D) TZM-bl cells were transfected with 1.0 μg of Tat and treated the next day with DMSO, 1 or 10 μM of 9AA, 2AA, 4AA, AH, 4AQ, or 100 nM flavopiridol. Cells were processed 48 hours post drug treatment for luciferase assays. Assays were performed in duplicate and an average value is shown.

We have previously observed that 9AA inhibits viral replication [46]. We were therefore interested if 9AA could specifically inhibit Tat dependent transcription. To test this hypothesis, CEM cells were transfected with HIV LTR-CAT with and without Tat. Two hours after transfection, the cells were treated with various concentrations of 9AA or 100 nM of flavopiridol as a positive control for transcription inhibition. Cells were harvested 48 hours post treatment and CAT assays performed. As expected, Flavopiridol treatment decreased viral transcription (Figure 1A, lane 7). Interestingly, 9AA treatment also decreased viral transcription in a dose dependent manner (lanes 3–6). Based on this data we can estimate that the IC_50 _of viral LTR Tat activated transcription inhibition by 9AA is approximately 250 nM. These results suggest that the observed inhibition of viral replication could be due at least in part to the ability of 9AA to inhibit viral transcription.

To determine if viral transcription inhibition also occurred on a fully chromatinized promoter, we utilized TZM-bl cells, which have an integrated LTR-luciferase reporter construct. TZM-bl cells were transfected with Tat and treated with various concentrations of 9AA or 100 nM flavopiridol the following day. Luciferase assays revealed that 9AA inhibited LTR transcription in a dose dependent manner, with 1 μM showing greater than 50% inhibition and 2.5 μM showing complete transcriptional inhibition (Figure 1B). We were interested if 9AA derivatives would display similar transcriptional inhibition ability. Four commercially available 9AA derivates were purchased (Figure 1C). Compounds 2-aminoacridine (2AA) and 4-aminoacridine (4AA) differ from 9AA only in the location of the amino moiety, while acridine hydrochloride (AH) lacks the amino group. 4-aminoquinoline (4AQ) retains the amino moiety in the same position as 9AA, but lacks the third aromatic ring. Luciferase assays performed with these compounds indicated that the presence and location of the amino moiety is critical for the activity of 9AA, as both AH and 4AA did not inhibit HIV-1 transcription (Figure 1D). Surprisingly, cells treated with 2AA exhibited an increase in viral transcription at both 1 μM and 10 μM. 4AQ treated cells showed limited viral transcription inhibition at 1 μM and approximately 50% inhibition at 1 μM. These results demonstrate the importance of both the presence and location of the amino moiety for the activity of 9AA and also show that, while improving 9AA activity, the third aromatic ring is not essential. The inhibition demonstrated by 9AA is specific to this class of compounds, as other amino acridines did not display activity to the same extent. In addition, they demonstrate that 9AA inhibits HIV-1 transcription in a specific manner.

### 9AA inhibits viral replication in cells with mutant p53

**Figure 2:**
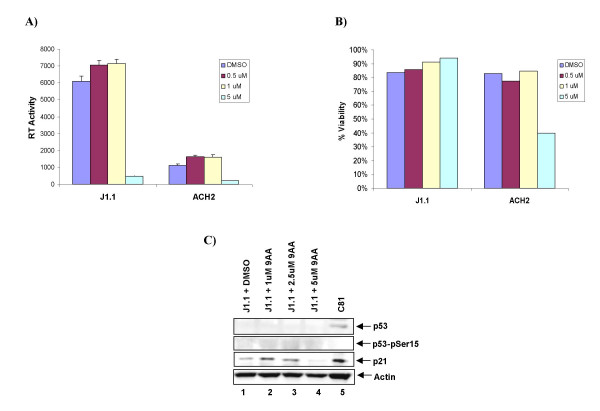
**9AA inhibits viral replication in cells with mutant p53**. HIV-1 infected (J1.1 and ACH2) T-cells were treated with 0.5, 1, and 5 μM of 9AA. A) RT activity was determined by at seven day post treatment. B) Cell viability was determined by trypan blue staining (~100/sample) seven days post treatment. C) J1.1 cells were treated with DMSO, 1, 2.5, and 5 μM 9AA and collected after 48 hours. Western blots were performed with anti-p21WAF1, anti-p53, anti-p53-phospho-S15, and actin antibodies.

To determine the contribution of p53 to 9AA mediated viral inhibition, viral replication was assessed in two different HIV-1 infected cell lines, J1.1 and ACH2, which vary in their p53 status. While there has been a discordance in the literature regarding p53 status in CEM cells [48,49], which is the parental cell line of ACH2; in our hands we have observed p53 DNA binding and activation of downstream signals, therefore p53 is functional in these cells. Conversely, Jurkat cells (parental cell line of J1.1) are well accepted as being p53 mutant cells, with R196X, T256A, D259G, and S260A mutations [49]. Both of these cells produce little virus in the absence of stimulation and therefore, supernatants were collected seven days after treatment to measure viral replication. RT assays indicated that viral replication was inhibited in both J1.1 and ACH2 cells at 5.0 μM 9AA (Figure 2A). Cell viability at seven days was unaffected in J1.1 cells, but decreased by approximately 50% in ACH2 cells (Figure 2B). These results suggest that the influence of 9AA on cellular viability may be dependent on the status of p53 and/or cell type dependent. We next examined the levels of p21WAF1 in J1.1 cells upon 9AA treatment. Figure 2C indicates that even though there is no detectable p53 in J1.1 cells, p21WAF1 is still induced with low levels of 9AA, which is similar to the results observed with ACH2 cells [44]. These results are interesting as they indicated that 9AA is capable of inhibiting viral replication in p53 mutant cells and that p21WAF1 is still induced by 9AA in the absence of functional p53.

### 9AA does not inhibit proliferation in uninfected cells

**Figure 3:**
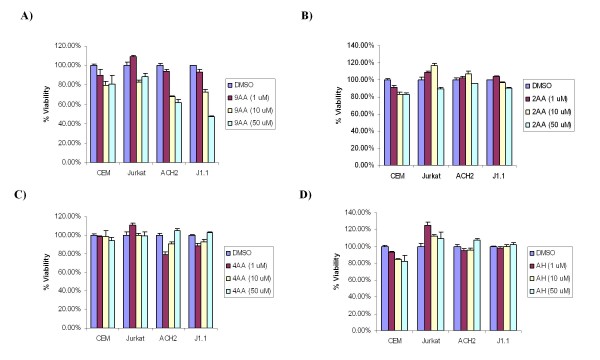
**9AA does not inhibit proliferation of uninfected cells**. Uninfected (CEM and Jurkat) and HIV-1 infected (ACH2 and J1.1) T-cells were treated with DMSO, 1, 10, or 50 μM of A) 9AA, B) 2AA, C) 4AA, D) AH. Cell proliferation/viability was determined by MTT assays. Treatments were performed in triplicate and samples analyzed at 48 hours.

As HIV-1 replicates more efficiently in proliferating cells and inhibiting cellular proliferation of uninfected cells could result in toxicity, we accessed whether 9AA treatment could be inhibiting cell proliferation. To this end, MTT assays were performed (Figure 3). MTT assays measure the reduction of the yellow tetrazolium MTT reagent (3-(4, 5-dimethylthiazolyl-2)-2, 5-diphenyltetrazolium bromide). Reduction occurs only by metabolically active cells and thus a reduced reading indicates decrease proliferation and possibly reduced viability. MTT assays were performed on uninfected and infected cells treated with either 9AA (panel A), 2AA (panel B), 4AA (panel C), or AH (panel D). Results indicate that up to 50 μM of 9AA had little to no effect on uninfected cells, but decreased cellular proliferation at 10 and 50 μM was observed for HIV-1 infected cells. It is important to note that low 9AA concentrations have been used consistently to observed viral inhibition, which is below the level at which cellular proliferation is being inhibited. Interestingly, all of the 3-ring 9AA derivatives had no effect on cellular proliferation in either uninfected or infected cells (panels B-D). Therefore, these results indicate that 9AA does not inhibit cellular proliferation in uninfected cells. These results also confirm that the inhibition of proliferation observed in the infected cells is dependent on the presence and location of the amino moiety of 9AA.

### 9AA induces AKT activity and stabilization of p21WAF1

**Figure 4:**
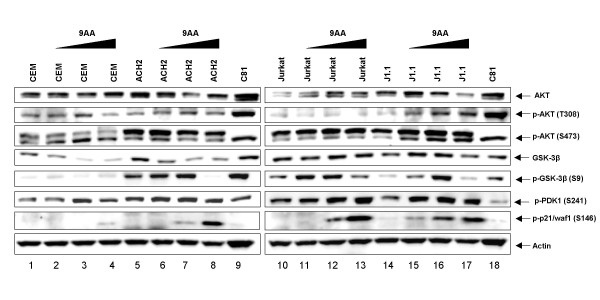
**9AA induces AKT activity and stabilization of p21WAF1**. Uninfected (CEM and Jurkat) and HIV-1 infected (ACH2 and J1.1) T-cells were treated with DMSO, 1, 2.5, or 5 μM of 9AA and collected 48 hours later. Western blot analysis was performed for Akt (pan), phospho-Akt (S473), phospho-Akt (T308), GSK3-β (pan), phospho-GSK3-β (S9), phospho-PDK1 (S241), phospho-p21WAF1 (S146), and actin.

Recently, Guo *et al*. demonstrated downregulation of p110γ, the catalytic subunit of the phosphoinositide 3-kinase (PI3K) family upon 9AA treatment of renal carcinoma cells [47]. Follow-up studies indicated that AKT and mammalian target of rapamycin (mTOR) signaling were inhibited, which contributed to p110γ downregulation, and possibly p53 and NF-κB alterations. Therefore, we were interested to see if similar events were occurring upon 9AA treatment in HIV-1 infected cells. Surprisingly, we observed a varying increase in AKT phosphorylation at both T308 and S473 in either ACH2 and J1.1 cells following 9AA treatment (Figure 4), indicating that AKT was activated upon 9AA treatment. C81 HTLV-1 infected cells are used as a positive control as AKT is active and phosphorylated in HTLV-1 infected cells in a Tax dependent manner [50]. These phosphorylation events were especially pronounced in J1.1 cells as compared to uninfected Jurkat cells (compare lanes 10 and 13 to lanes 14 and 17). AKT is phosphorylated at both T308 and S473 to induce enzyme activation, with T308 being phosphorylated by PDK1 [51,52] and S473 by mTOR [53-56]. To confirm that AKT was activated we examined a downstream substrate of AKT, GSK3-β [57]. GSK3-β is phosphorylated by AKT on S9, inhibiting the activity of GSK3-β and regulating downstream events such as glycogen synthesis and β-catenin signaling [58,59]. Total GSK3-β levels remained constant in Jurkat and J1.1 cells, but decreased upon 9AA treatment in ACH2 and CEM cells. Interestingly, p-GSK3-β levels were increased in Jurkat, J1.1 and ACH2 cells, while CEM cells display no p-GSK-3β with or without 9AA treatment. We next examined an upstream event of AKT activation, PDK1 phosphorylation at S241, which is an autophosphorylation event necessary for PDK1 activation [60]. PDK1 exhibited increased phosphorylation and thus activation in J1.1 cells, but only a modest increase in CEM, ACH2, and Jurkat cells. Finally, we examined p21WAF1 phosphorylation on S146, which has been shown to be phosphorylated by AKT, resulting in increased p21WAF1 stability [61]. Both ACH2 and J1.1 cells displayed p21WAF1 phosphorylation following 9AA treatment. Collectively these results indicate that AKT activity is increased following 9AA treatment, resulting in increased p21WAF1 phosphorylation, which could aid in stabilization of p21WAF1. This phosphorylation was observed in both p53 wildtype and p53 mutant cells.

### p21WAF1 binds to cyclin T and cdk9 *in vitro*

**Figure 5:**
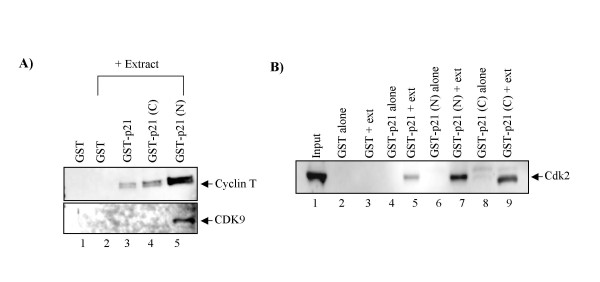
**p21WAF1 binds to cyclin T and cdk9 *in vitro***. One μg of GST, GST-p21, GST-p21 (C), or GST-p21 (N) were added to 1 mg of CEM cell lysates and allowed to bind overnight. The next day, complexes were bound to glutathione sepharose beads, washed, and analyzed by SDS-PAGE, followed by western blotting for cyclin T and cdk9 (Panel A) or cdk2 (Panel B). ext = Extract.

As 9AA treatment resulted in increased p21WAF1 expression and stability, we were interested to determine if p21WAF1 could bind to the cyclin T/cdk9 complex since this is one of the key factors regulating HIV-1 promoter activity. To this end, we performed a GST-pulldown assay. GST, GST-p21, GST-p21 C-terminus [GST-p21 (C)], and GST-p21 N-terminus [GST-p21 (N)] were incubated with CEM cellular extracts overnight at 4°C. The next day, complexes were bound to glutathione sepharose beads, washed with TNE buffer, analyzed by SDS-PAGE, and western blotted with anti-cyclin T and cdk9 antibodies. Full length p21WAF1 as well as the C- and N-terminal regions of p21WAF1 were observed in complex with cyclin T (Figure 5A). The strongest binding was observed with the N-terminal region. Interestingly, the N-terminal region of p21WAF1 was also observed in complex with cdk9. This data is in agreement with other studies indicating that the N-terminal of p21WAF1 contains cyclin and cdk binding domains, whereas the C-terminal only contains a cyclin binding site [62,63]. As a positive control, we also tested for cdk2 and p21WAF1 binding (Figure 5B). Therefore, these results suggest that p21WAF1 is a component of the p-TEFb complex.

### 9AA treatment results in loss of cdk9 from the viral LTR

**Figure 6:**
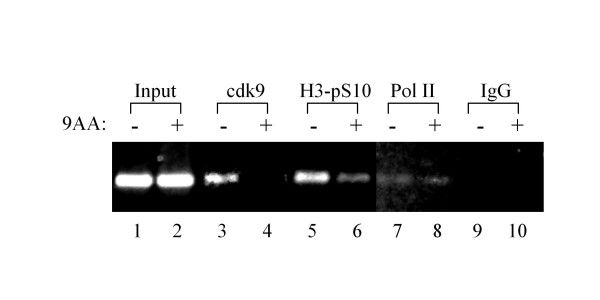
**9AA treatment results in loss of cdk9 from the HIV-1 LTR**. ACH2 cells were treated with either DMSO or 2.5 μM 9AA for 48 hours, cross-linked and collected for ChIP analysis. Antibodies against cdk9, histone H3-phospho-Ser10 (H3-pS10), RNA Polymerase II (Pol II), and rabbit IgG were utilized.

Due to the observed binding of p21WAF1 with the pTEFb complex and the importance of cdk9 in HIV-1 transcription, we performed ChIP experiments to determine if cdk9 binding at the LTR was altered upon 9AA treatment. ACH2 cells were treated with either DMSO or 9AA and collected 48 hours later. Results indicated that 9AA treatment results in a dramatic loss of cdk9 from the LTR (Figure 6). In addition, we observed a reduction in the levels of histone H3-phospho-S10 levels upon 9AA treatment. These results suggest that 9AA treatment alters LTR binding proteins to induce transcriptional inhibition.

## Discussion

In this study we have demonstrated that 9AA is an HIV-1 transcriptional inhibitor that acts without inducing cell death or inhibiting cellular proliferation of uninfected cells. Interestingly, we observed 9AA inhibition of HIV-1 replication in both p53 wildtype and p53 mutant cells. However, in both cell types p21WAF1 levels were increased following 9AA treatment. Unexpectedly, we found increased AKT activity upon 9AA treatment as well as phosphorylation of p21WAF1 at S146, a known target of AKT, which induces p21WAF1 stability. We also observed p21WAF1 in complex with cdk9/cyclin T *in vitro*, suggesting that p21WAF1 may act as an inhibitor of cdk9/cylin T1 kinase activity. Finally, we found that cdk9 was removed from the viral LTR following 9AA treatment, indicating one mechanism for loss of viral transcription.

The interplay between p53 and HIV-1 is of significant interest to the HIV-1 field. Specifically, p53 and Tat antagonize each other, resulting in inhibition of Tat transcription by p53 and downregulation of p53 dependent transcription by Tat [21]. In addition, the activation of p53 is known to induce apoptosis in response to gp120 [2,64,65], where cell death can be induced by through mTOR-mediated phosphorylation of p53 on S15 and subsequent phosphorylation of p53 on S46 [65,66]. p53 phosphorylation on S15 was observed following 9AA treatment, however cell death was not observed at low levels of compound treatment. S15 phosphorylation is a priming event necessary for other p53 post-translational modifications, with the end result of p53 activation. Therefore, our results indicate that 9AA treatment activates p53 and inhibits HIV-1 without inducing apoptosis. Conversely there has also been data indicating that knockdown of p53 through RNA interference results in a marked reduction in Tat-induced transcription [67]. One potential explanation for the discrepancy is that the above mentioned study examined acutely infected cells, whereas many of the other investigators used chronic or latently infected models, where the status of p53 is unknown. In addition, our current results point toward both p53 dependent and p53 independent activation of p21Waf1 by 9AA treatment. We believe that p21Waf1 may be the key protein in regulating cyclin/cdk complexes in these chronically or latently infected cells.

Recently, Zhang *et al*. investigated p21WAF1 as a potential molecular barrier for HIV-1 infection of stem cells [68]. Hematopoietic stem cells were previously demonstrated to be highly resistant to HIV-1 infection [69-71]. In this study, p21WAF1 was revealed to restrict HIV-1 infection in primitive hematopoietic cells. By knockdown of the endogenous p21WAF1 levels using siRNAs, the stem cells became highly susceptible to HIV-1 infection. Further, it was shown that the effect of p21WAF1 is specific as the silencing of other p21WAF1 related proteins, p27 and p18 had no effect on HIV-1 infection. Based on these results it was suggested that p21WAF1 may be a possible restriction factor, like TRIM5 and APOBEC3G genes [72-76]. Interestingly, previous research showed that high-titer infection of HIV-1 in T lymphocytes resulted in a loss of the endogenous p21WAF1 [27], further demonstrating the importance of p21WAF1 in HIV-1 biology.

Our results indicate that p21WAF1 can be induced upon 9AA treatment independently of p53. In fact, p21WAF1 can be induced by a wide array of transcription factors independent of p53 including Sp1/Sp3, BRCA1, E2F-1/E2F-3, Smad3/4, STAT1, STAT3, STAT5, C/EBPα, and C/EBPβ [77]. In addition, there are a number of transcription factors that are involved in the repression of p21WAF1 transcription including c-myc, c-jun, and Id1 [78]. A number of theses factors also have an influence on HIV-1 transcription, including Sp1, C/EBPs, and c-myc [79]. Therefore future studies will be focused on identifying signaling pathways that are altered upstream of p21WAF1 induction following 9AA treatment.

Surprisingly we observed an increased in AKT activity and GSK3-β phosphorylation following 9AA treatment in both p53 wildtype and p53 mutant cells. At first glance these results seemed puzzling; however there are a number of mechanisms that could explain this observation. MDM2 is a p53 transcriptional target that also functions in a feedback loop to regulate p53 levels through inducing p53 proteasomal degradation [80-84]. In order for MDM2 to target p53 to the proteasome it must be phosphorylated within its central domain [85]. Interestingly, MDM2 is phosphorylated by GSK3, resulting in decreased p53 stability [86]. In addition, Boehme *et al*. found that p53 is stabilized following DNA damage due to DNA PK mediated activation of AKT, phosphorylation and inhibition of GSK3, and consequently inhibition of MDM2 [87]. Therefore in p53 wildtype cells, AKT activation could serve to increase the stability of p53 through the downstream inhibition of MDM2. In both p53 wildtype and mutant cells we observed p21WAF1 phosphorylation at S146, which has been shown to enhance stability of p21WAF1 as well as disrupt the interaction of p21WAF1 with PCNA [61,88]. S146 phosphorylation was originally described as an AKT event [61], but can also be induced by PKC which is downstream of AKT [89]. Hela cells treated with siRNA against PKCδ display reduced p21WAF1 stability [89]. Finally, mTOR has been shown to phosphorylate S15 of p53 [64,65], which is enhanced following 9AA treatment. Our results demonstrate that two substrates of mTOR, p53 S15 and AKT S473 display increased phosphorylation following 9AA treatment. Collectively, these results suggest that AKT and mTOR are activated following 9AA treatment and may help stabilize p53-p21WAF1 activation.

**Figure 7:**
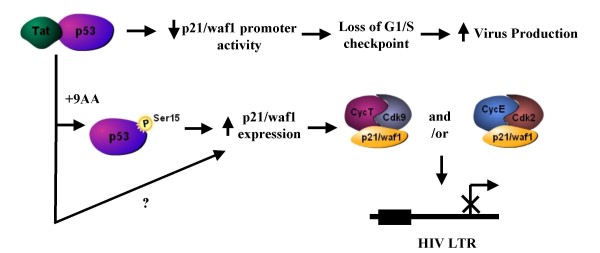
**Model of 9AA induced activation of p21WAF1 in HIV-1 infected cells**. In HIV-1 infected cells, p53 is inactivated through the binding to Tat. Upon 9AA treatment, p53 becomes phosphorylated at Ser15, resulting in loss of Tat binding and transcription of p53 dependent promoters, specifically p21WAF1. p21WAF1 protein levels are increased and this newly formed p21WAF1 is able to bind to both cycT/cdk9 and cycE/cdk2 complexes. Binding of p21WAF1 to cyclin/cdk complexes results in inhibition of HIV-1 LTR transcription possibly through decreased RNA Pol II and/or histone H1 phosphorylation. p21WAF1 can also be activated by 9AA treatment in p53 mutant cells by a yet undefined mechanism.

Cyclin T/cdk9 are key factors regulating HIV-1 promoter activity, forming the main components of the p-TEFb complex. p-TEFb, the positive elongation factor, is critical for both transcriptional initiation and elongation, through the phosphorylation of RNA polymerase II (RNA Pol II) C-terminal domain (CTD) [90-99]. p-TEFb is a multi-protein complex that can be found in two distinct complexes, a small highly active p-TEFb that contains cdk9 with a cyclin partner (cyclin T1, T2a, T2b, or K), and a large inactive p-TEFb complex [100-103]. Components of the large complex, 7SK small nuclear RNA (snRNA) and the hexamethylene bisacetamide-induced protein 1 (HEXIM1), have been shown to inhibit p-TEFb activity [100,102,104,105]. Most cdks are inhibited by either the INK (p15, p16, p18, and p19) or Cip/Kip (p21WAF1, p27, p57) family of cdk inhibitors [31,106-112]. To date, cdk9 has not been shown to be inhibited by either cdk inhibitor family. Here for the first time, we demonstrate p21WAF1 binding to cdk9 and cyclin T *in vitro*. It is possible that p21WAF1 needs to be in a phosphorylated state to allow binding to cdk9/cylin T as was observed following 9AA treatment. Indeed, it is known that phosphorylation of p21WAF1 influences its binding partners, with phosphorylation of S146 disrupting binding to PCNA [88]. It remains to be seen whether phosphorylation of p21WAF1 increases the binding to cdk9, cyclin T1, or to both, as p21WAF1 interacts with both cdk and cyclin partners through different motifs [62]. p21WAF1 has a short half life of approximately one hour, therefore phosphorylation of S146 could also influence the formation of a p21WAF1-p-TEFb complex through stabilizing p21WAF1. We hypothesize that p21WAF1 inhibits cyclin T/cdk9 activity resulting in inhibition of RNA Pol II CTD phosphorylation and HIV-1 transcription. Along these lines, following 9AA treatment we observed loss of cdk9 at the viral promoter. We have also previously shown that 9AA treatment inhibits cyclin E/cdk2 kinase activity [46]. Therefore, a combination of cyclin E/cdk2 and cyclin T/cdk9 inhibition by increased p21WAF1 levels would severely diminish the levels of phosphorylated RNA Pol II CTD and inhibit HIV-1 transcription (Figure 7).

Finally, we have begun to dissect particular structural features that are critical for 9AA's mechanism of action through the use of 9AA derivatives. Our studies have shown that the amino moiety is critical for transcriptional inhibition as loss or movement of this moiety results in loss of 9AA activity. Furthermore, removal of the third aromatic ring of 9AA only partially diminishes 9AA's activity, indicating that it is not a critical structural feature. Future studies will further define the structure activity relationships of this class of molecules in search for a more potent and specific transcriptional inhibitor.

## Competing interests

The authors declare that they have no competing interests.

## Authors' contributions

IG performed western blot analysis, GST-pulldowns, and luciferase assays. RE performed luciferase assays. EA performed drug treatment studies. LC performed ChIP experiments. RD and WC performed western blot analysis. CD provided essential advice in regards to the 9AA derivatives to test. FK aided in the preparation of the manuscript and in experimental design. KK coordinated the research and experimental design, as well as wrote the manuscript.
